# Supplementary figures and images for: Coordinated circadian timing through the integration of local inputs in Arabidopsis thaliana
Source: PLoS Biol. 2019 Aug 15;17(8):e3000407. doi: 10.1371/journal.pbio.3000407 (PMC6695092; doi:10.1371/journal.pbio.3000407)

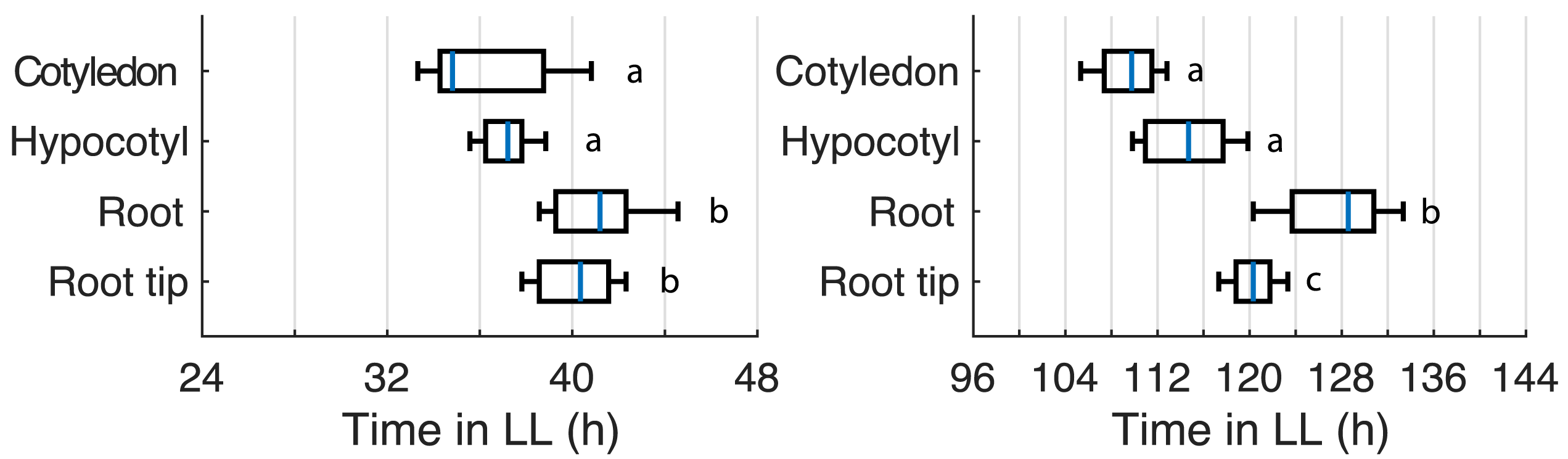

Supplement: S1 Fig — Times of peaks of GI::LUC expression in different organs during the first (left) and final (right) observed oscillations under the LD-to-LL condition. Means are statistically different (p < 0.05, one-way ANOVA, Tukey post hoc tests) if they do not have a letter in common. Box plots indicate the median and upper and lower quartiles, and whiskers the 9th and 91st percentiles of organs scored as rhythmic. N and n are as presented in Fig 2. See S2 File for exact n, test statistics, and percentage rhythmicity of each organ. Underlying data are available from https://gitlab.com/slcu/teamJL/greenwood_etal_2019. GI, GIGANTEA; LD, light-dark; LL, constant light; LUC, LUCIFERASE. (TIF) [file pbio.3000407.s001.tif]

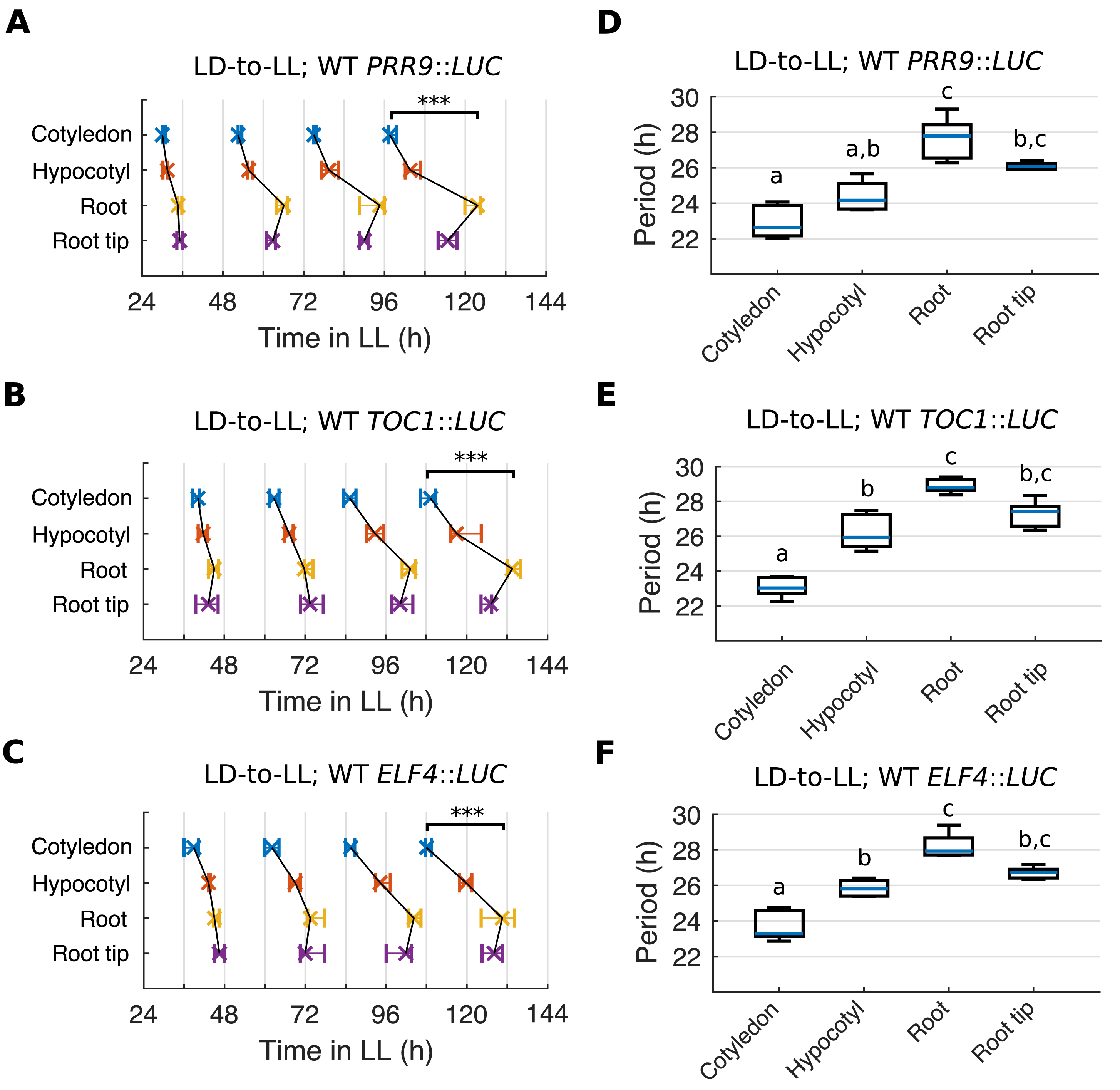

Supplement: S2 Fig — (A–C) Times of peaks of expression of PRR9::LUC (A), TOC1::LUC (B), or ELF4::LUC (C) in different organs under the LD-to-LL condition. Plots represent the 25th percentile, median, and the 75th percentile for the peak times of organs scored as rhythmic. ***p < 0.001, Kruskal-Wallis ANOVA. (D–F) Period estimates of PRR9::LUC (D), TOC1::LUC (E), or ELF4::LUC (F) expression for different organs imaged under the LD-to-LL condition. Box plots indicate the median and upper and lower quartiles, and whiskers the 9th and 91st percentiles of organs scored as rhythmic. For PRR9::LUC, N = 3; TOC1::LUC, N = 3; ELF4::LUC, N = 3. For all, n = 11–18. N represents the number of independent experiments and n the total number of organs tracked. See S1 and S2 Files for exact n, test statistics, and percentage rhythmicity of each organ. A subset of PRR9::LUC data is an analysis of time-lapse movies carried out in our previous work [5]. Underlying data are available from https://gitlab.com/slcu/teamJL/greenwood_etal_2019. ELF4, EARLY FLOWERING 4; LD, light-dark; LL, constant light; LUC, LUCIFERASE; PRR9, PSEUDO-RESPONSE REGULATOR 9; TOC1, TIMING OF CAB EXPRESSION 1. (TIF) [file pbio.3000407.s002.tif]

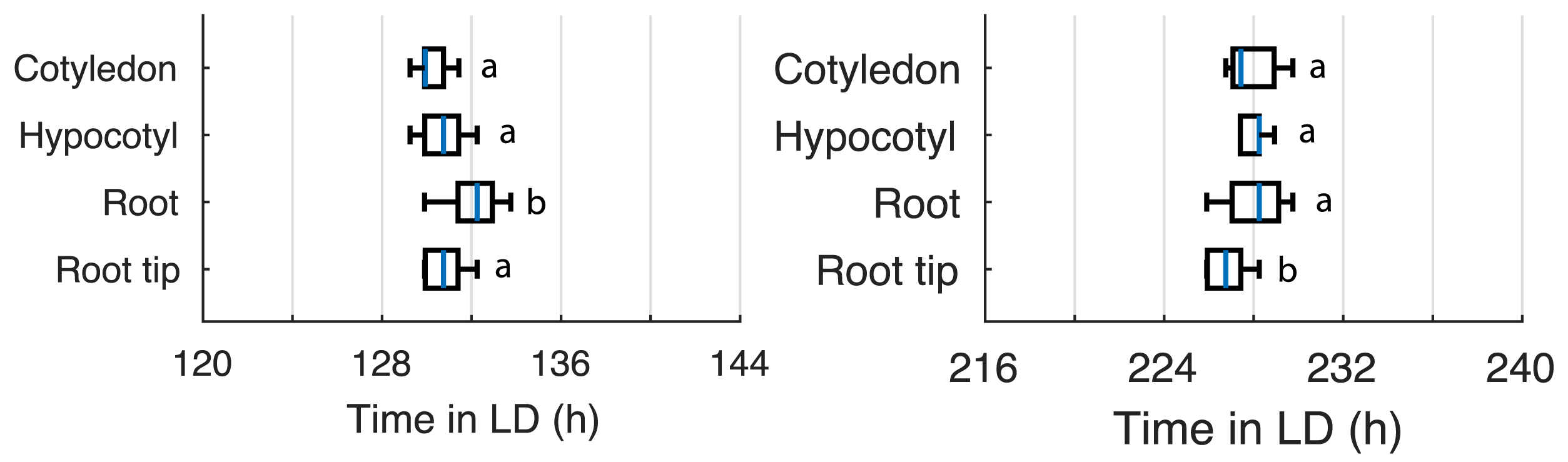

Supplement: S3 Fig — Times of peaks of GI::LUC expression in different organs during the first (left) and final (right) observed oscillations under the LD-to-LD condition. Means are statistically different (p < 0.05, one-way ANOVA, Tukey post hoc tests) if they do not have a letter in common. N and n are as presented in Fig 2. N represents the number of independent experiments and n the total number of organs tracked. See S2 File for exact n, test statistics, and percentage rhythmicity of each organ. Box plots indicate the median and upper and lower quartiles, and whiskers the 9th and 91st percentiles of organs scored as rhythmic. Underlying data are available from https://gitlab.com/slcu/teamJL/greenwood_etal_2019. GI, GIGANTEA; LD, light-dark; LUC, LUCIFERASE. (TIF) [file pbio.3000407.s003.tif]

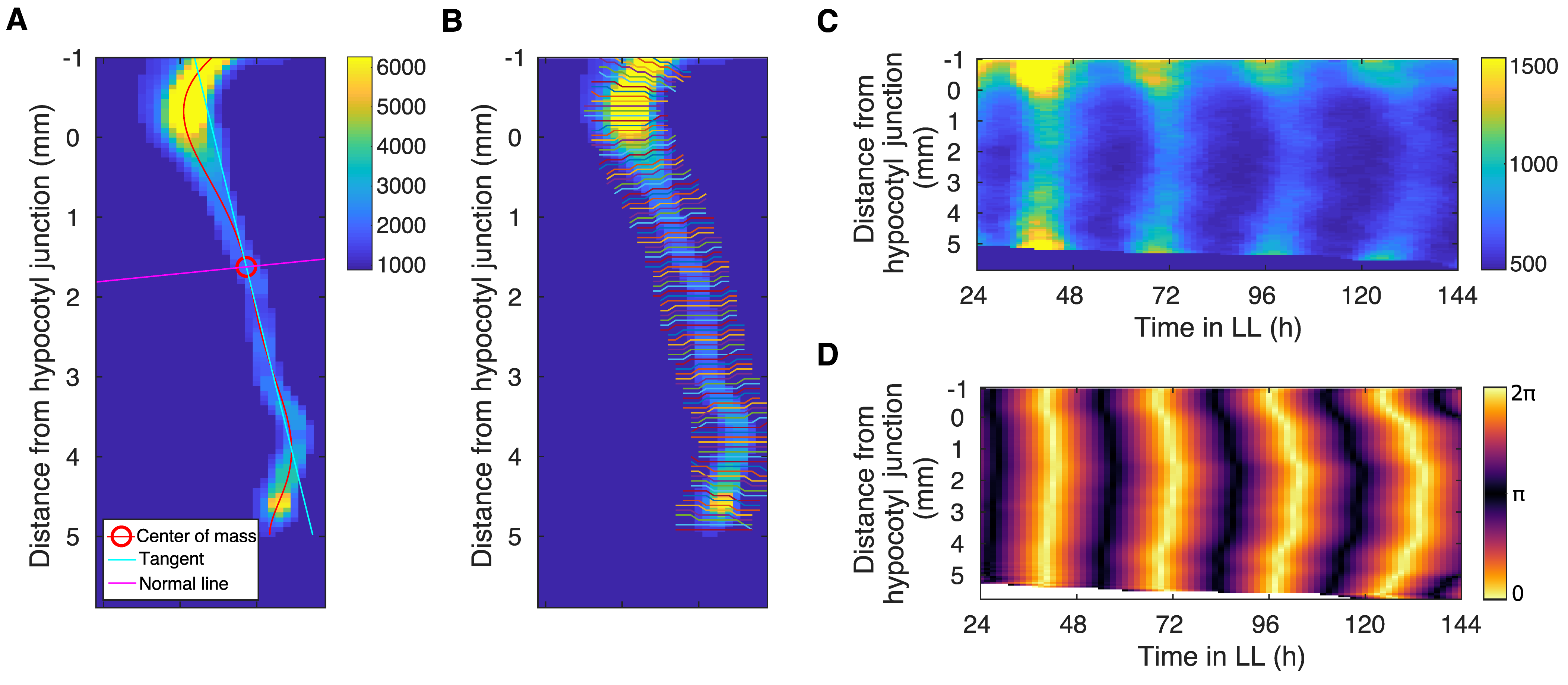

Supplement: S4 Fig — (A) Luciferase images are thresholded and a line fitted through the center of mass of the organ. At each index on this line, the normal line is taken. (B) Each normal line is rasterized and limited to 5 pixels around the center of mass to give pixel coordinates for longitudinal sections. (C) The mean value across longitudinal sections is taken at each time point to create a raw intensity space-time plot of a single seedling. (D) The phase of the oscillations is extracted using a wavelet transform to give a space-time map of the phase. (TIF) [file pbio.3000407.s004.tif]

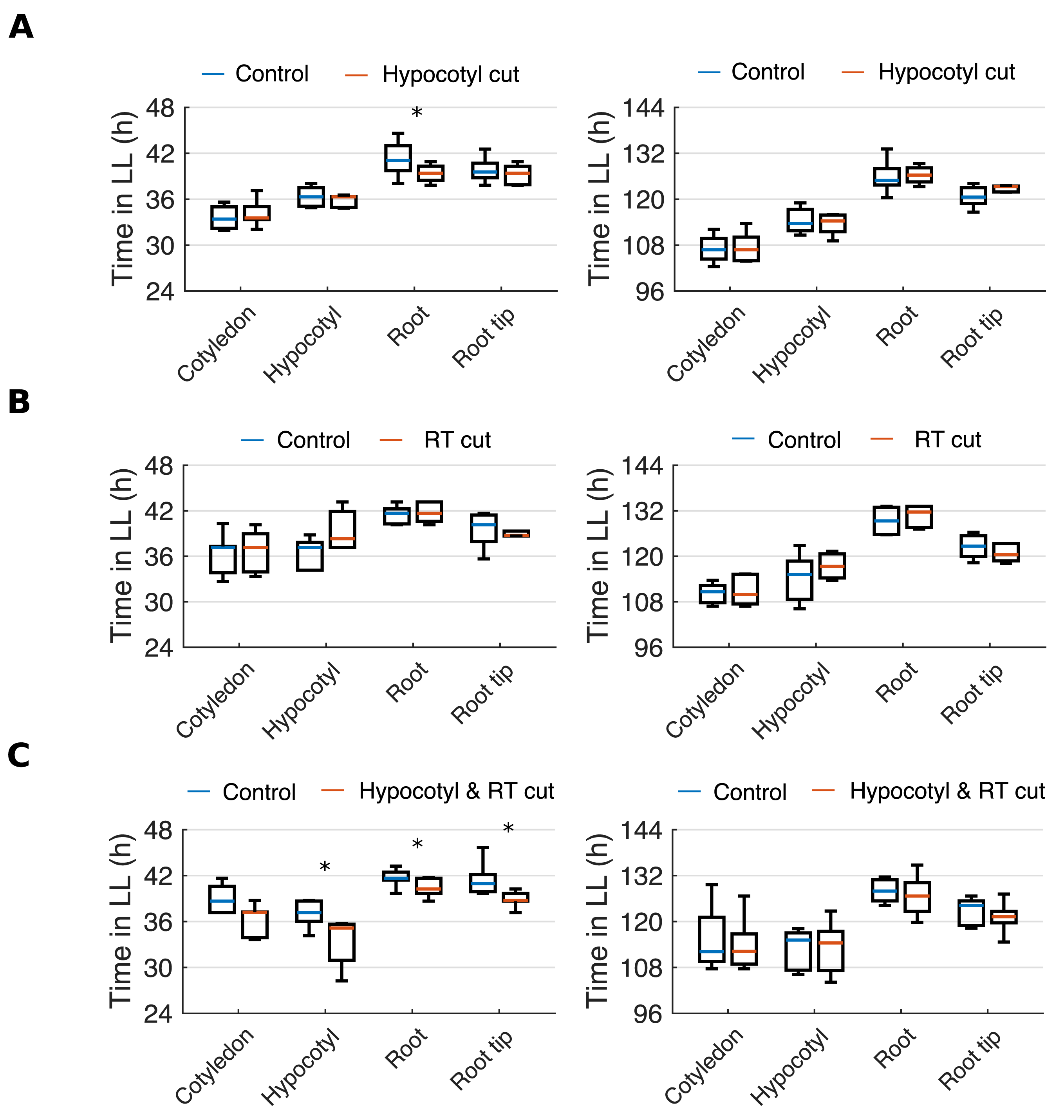

Supplement: S5 Fig — (A-C) Times of peaks of GI::LUC expression in different organs for the first (left) and final (right) observed oscillations following a cut at the hypocotyl junction (A), root tip (B), or both the hypocotyl junction and root tip (C) conditions. *p < 0.05, Wilcoxon rank-sum test. N and n are as in Fig 3. N represents the number of independent experiments and n the total number of organs tracked. See S2 File for exact n, test statistics, and percentage rhythmicity of each organ. Box plots indicate the median and upper and lower quartiles, and whiskers the 9th and 91st percentiles of organs scored as rhythmic. Underlying data are available from https://gitlab.com/slcu/teamJL/greenwood_etal_2019. GI, GIGANTEA; LUC, LUCIFERASE. (TIF) [file pbio.3000407.s005.tif]

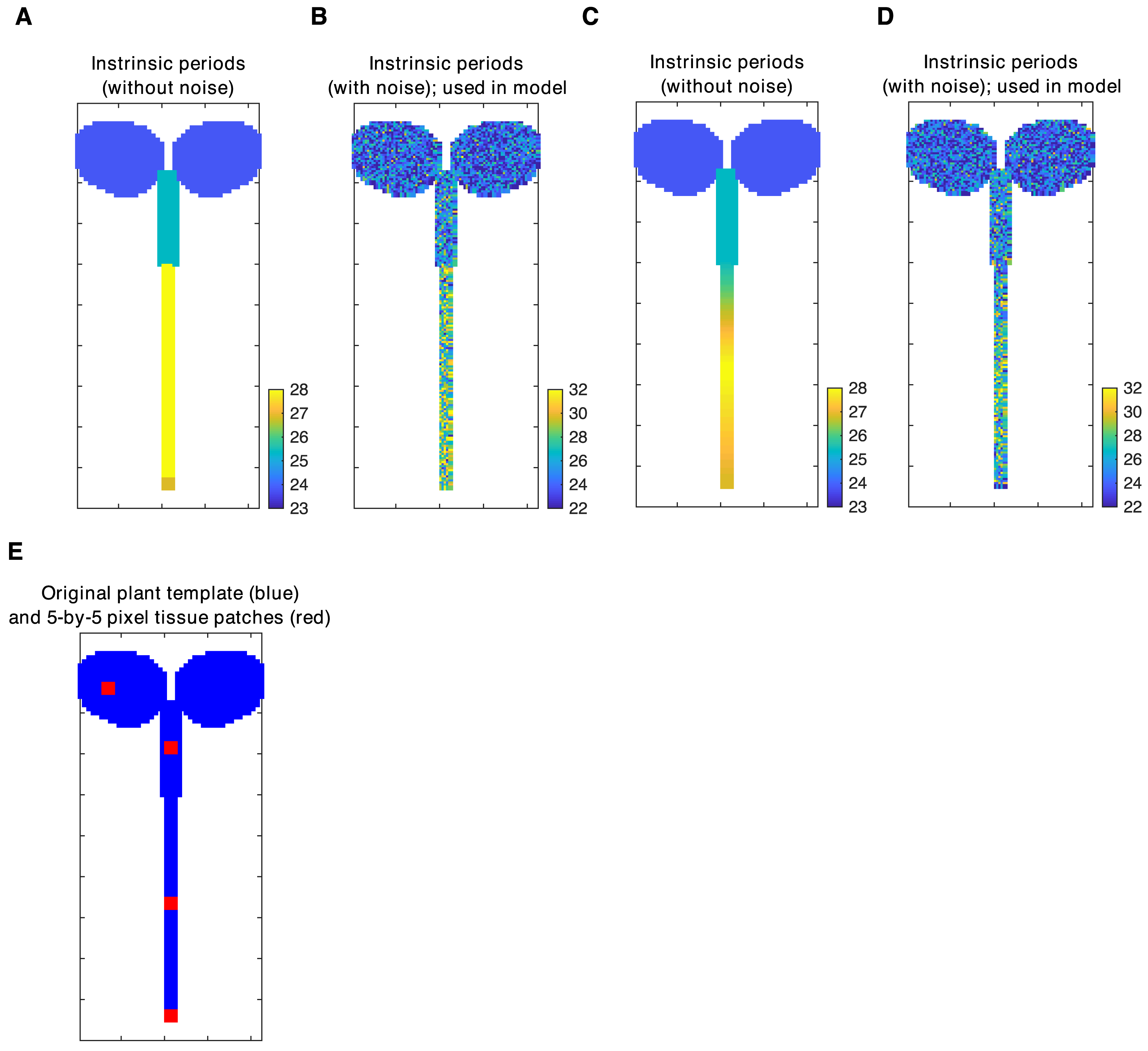

Supplement: S6 Fig — (A, B) Template for simulations, in which, in A, the periods of the pixels in each tissue are set to the mean periods measured in the LD-to-LL experimental data. In B, a representative set of periods for each region are shown, as drawn from the period distributions described in Materials and methods. (C, D) Template for simulations of the alternative model, in which, in C, periods of the pixels in each tissue are set to the mean periods measured in the LD-to-LL experimental data but with a gradient of periods in the root, as described in Materials and methods. In D, a representative set of seedling periods are shown, drawn from the period distributions and gradient described in Materials and methods. (E) The 5-by-5 pixel ROIs used for phase and period analyses are identified on the template. LD, light-dark; LL, constant light; ROI, region of interest. (TIF) [file pbio.3000407.s006.tif]

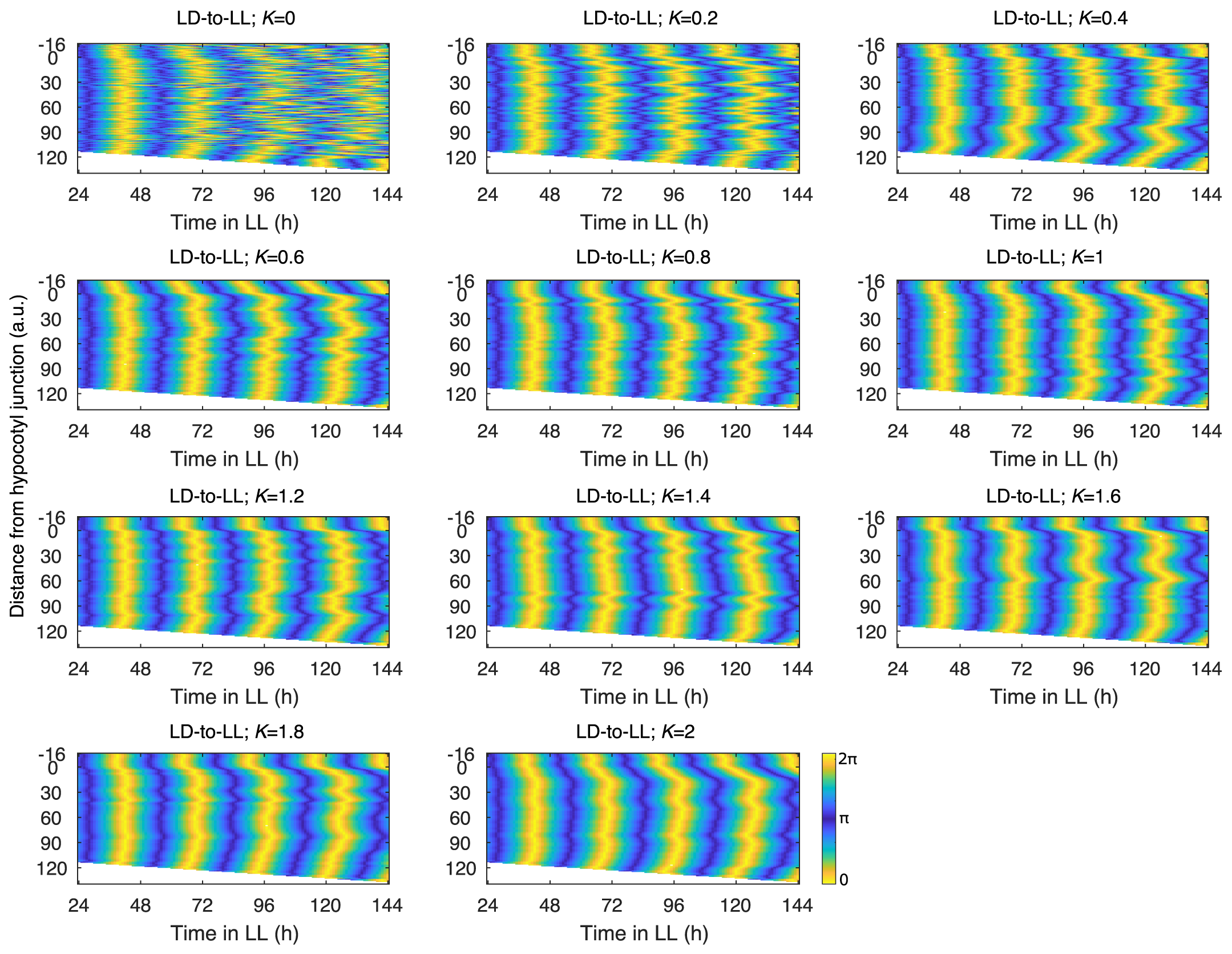

Supplement: S7 Fig — Phase plots of simulated GI expression across longitudinal sections of the hypocotyl and root. Each phase plot is a simulation of a single seedling, each with a different strength of coupling (K). GI, GIGANTEA; LD, light-dark; LL, constant light. (TIF) [file pbio.3000407.s007.tif]

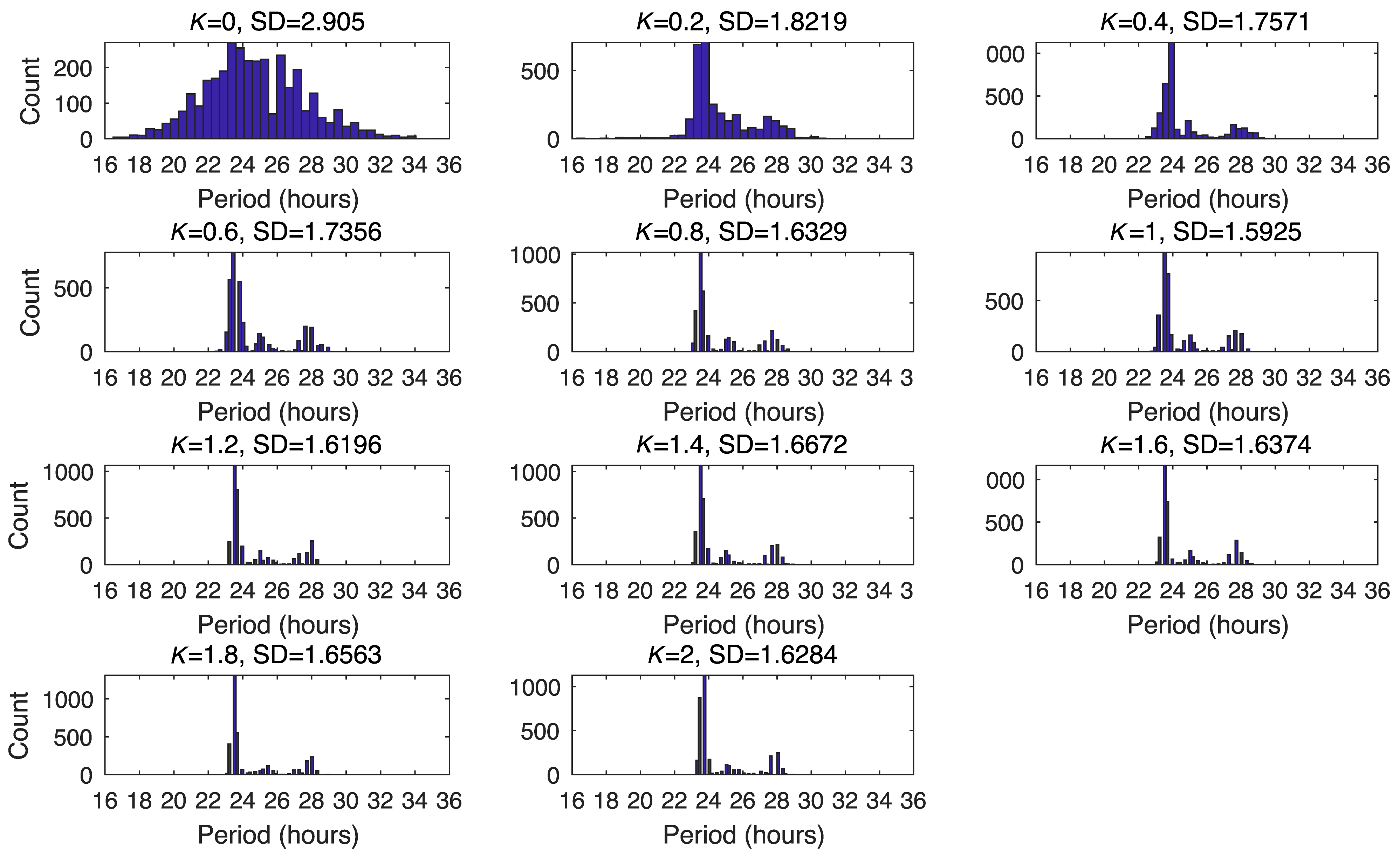

Supplement: S8 Fig — The distribution of periods of simulated GI from individual pixels on the model template of a seedling is shown. Each plot shows the distribution for the simulation of a single seedling under the LD-to-LL condition with different strengths of coupling (K). GI, GIGANTEA; LD, light-dark; LL, constant light; SD, standard deviation. (TIF) [file pbio.3000407.s008.tif]

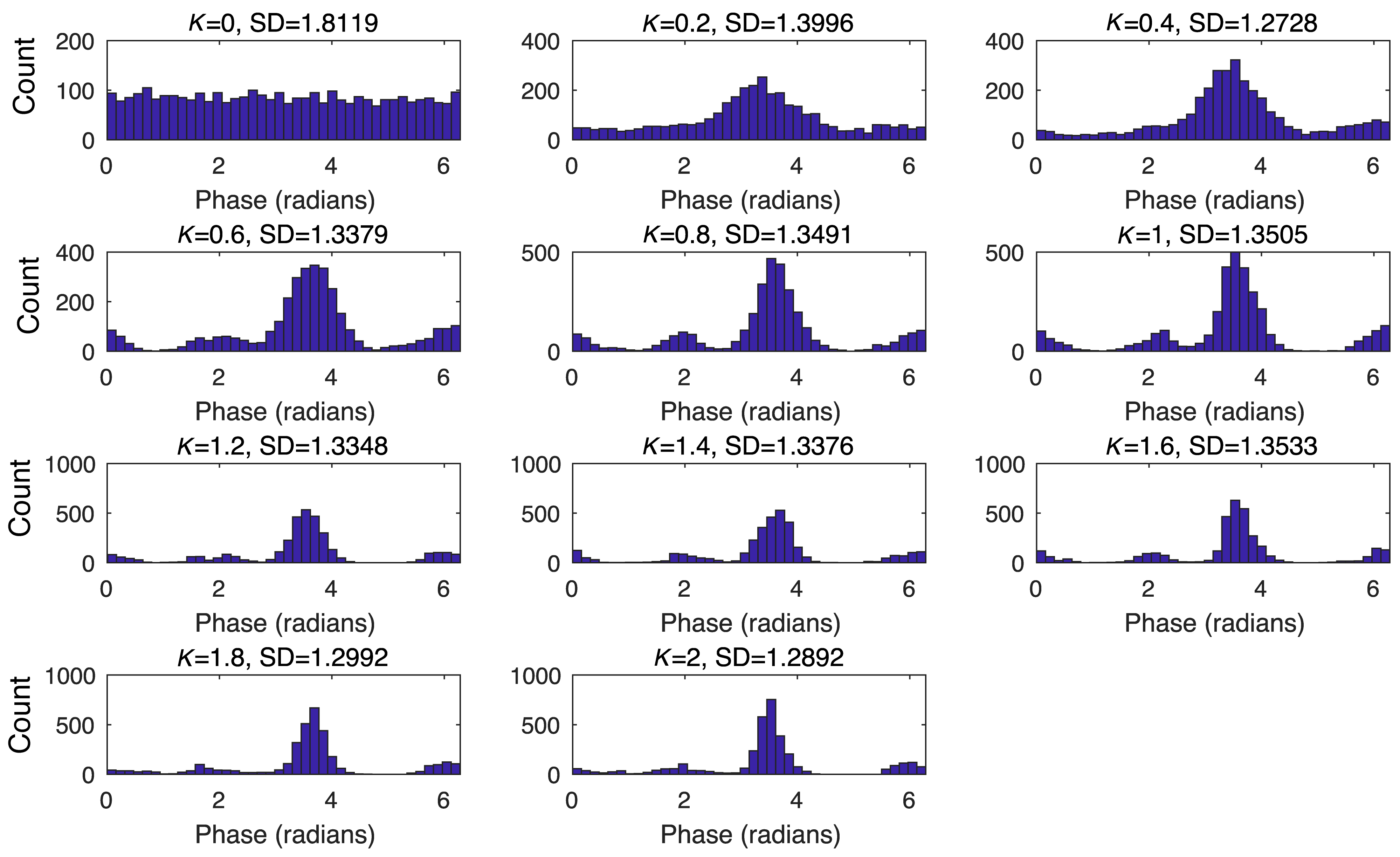

Supplement: S9 Fig — The distribution of phases of simulated GI from individual pixels on the model template of a seedling is shown. Each plot shows the distribution for the simulation of a single seedling under the LD-to-LL condition with different strengths of coupling (K). GI, GIGANTEA; LD, light-dark; LL, constant light; SD, standard deviation. (TIF) [file pbio.3000407.s009.tif]

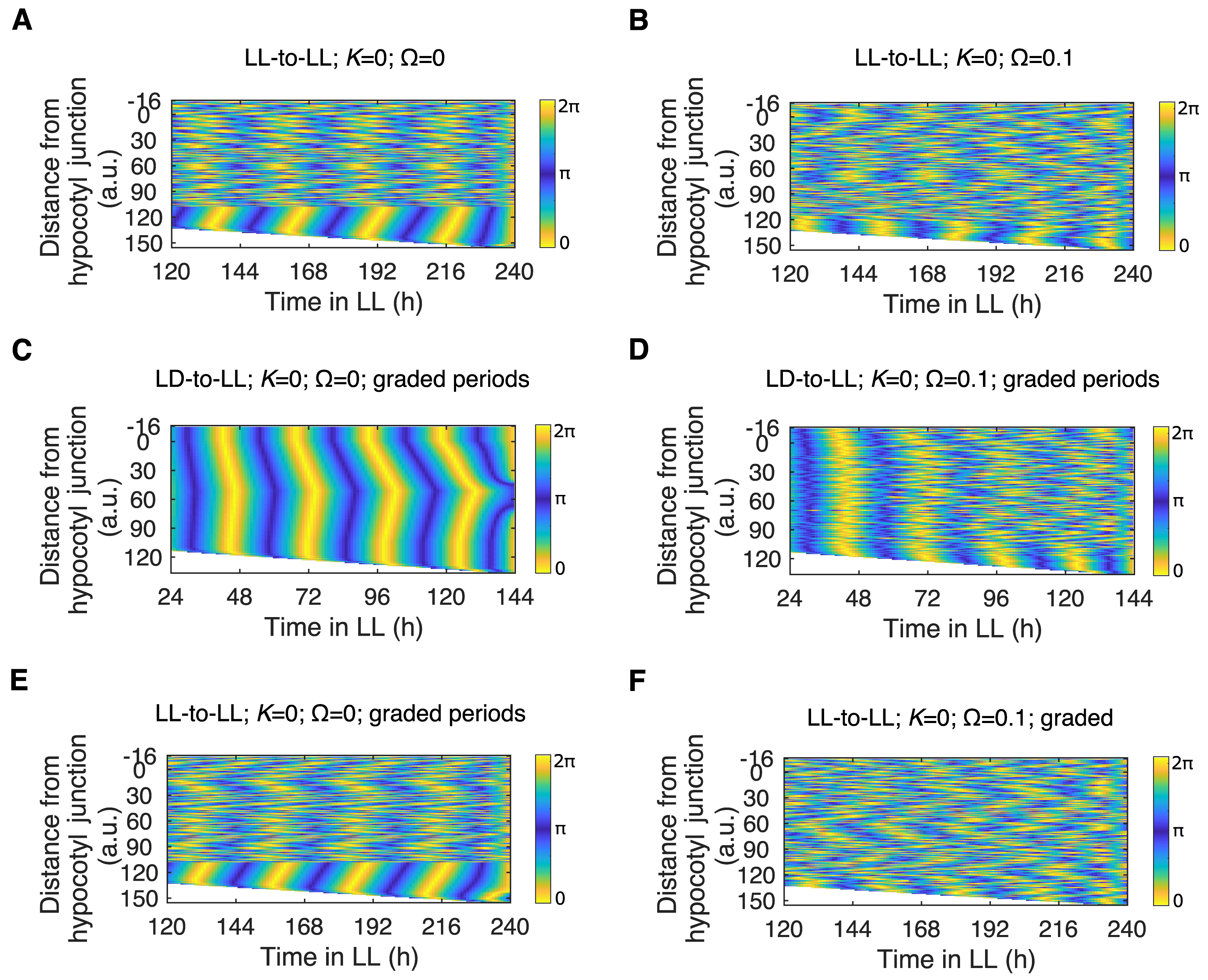

Supplement: S10 Fig — (A, B) Phase plot of simulated GI expression across longitudinal sections of the hypocotyl and root of a single seedling for the LL-to-LL condition in the absence of coupling (K = 0), but with period differences. In A, periods of the pixels in each tissue are set to the mean periods measured in the LD-to-LL experimental data, without noise (Ω = 0). In B, a representative set of periods for each region are shown, as drawn from the period distributions described in Materials and methods (Ω = 0.1). (C, D) Phase plot of simulated GI expression across longitudinal sections of the hypocotyl and root of a single seedling for the LD-to-LL condition in the absence of coupling (K = 0). In C, periods in the root region are graded with a maximum period in the middle of the root, without noise (Ω = 0). In D, periods are also graded in the root, but periods are drawn from a distribution (Ω = 0.1). See Materials and methods for details. (E, F) Phase plot of simulated GI expression across longitudinal sections of the hypocotyl and root of a single seedling for the LL-to-LL condition in the absence of coupling (K = 0). In E, periods in the root region are graded with a maximum period in the middle of the root, without noise (Ω = 0). In F, periods are also graded in the root, but periods are drawn from a distribution (Ω = 0.1). See Materials and methods for details. GI, GIGANTEA; LD, light-dark; LL, constant light. (TIF) [file pbio.3000407.s010.tif]

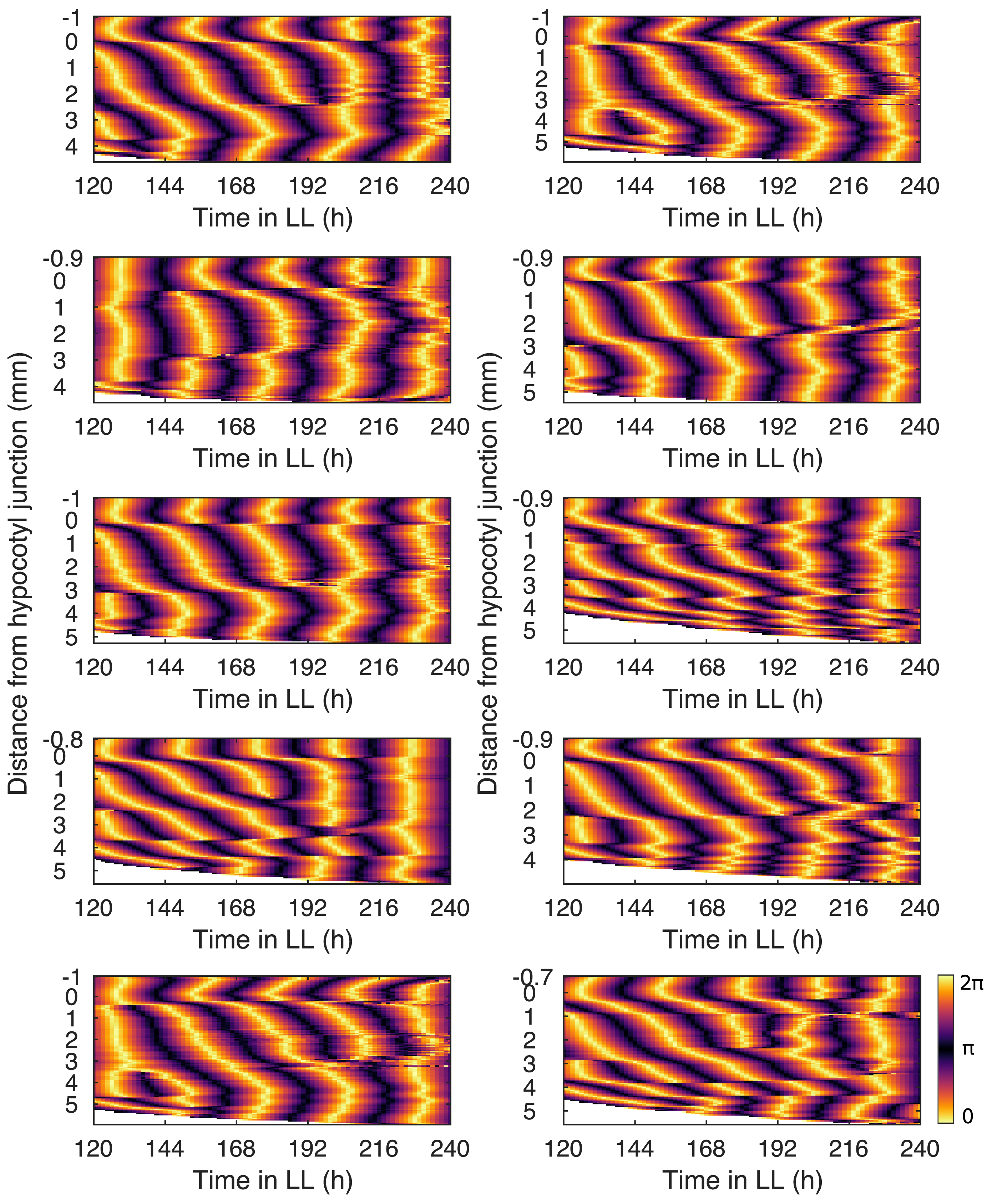

Supplement: S11 Fig — Phase plots of GI::LUC expression across longitudinal sections of the hypocotyl and root. Each phase plot is of a single seedling that is representative for the LL-to-LL condition. N and n are as presented in Fig 5. N represents the number of independent experiments and n the total number of organs tracked. See S1 or S2 Files for exact n and percentage rhythmicity. GI, GIGANTEA; LL, constant light; LUC, LUCIFERASE. (TIF) [file pbio.3000407.s011.tif]

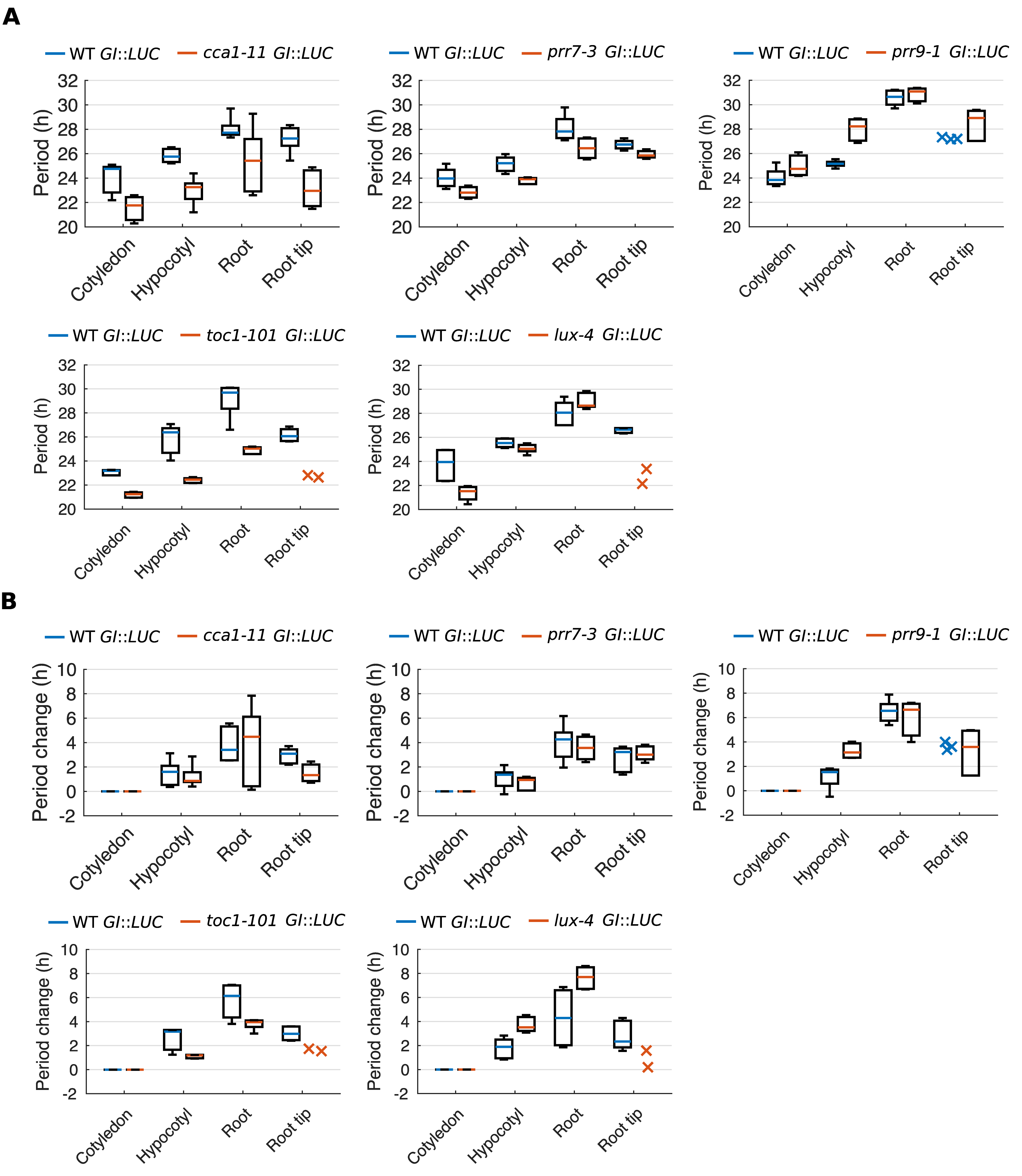

Supplement: S12 Fig — (A) Period estimates for GI::LUC expression from different organs imaged under the LD-to-LL condition in circadian mutant lines. (B) Period change relative to the cotyledon for GI::LUC expression from different organs imaged under the LD-to-LL condition in circadian mutant lines. For cca1-11, N = 4; prr7-3, N = 4; prr9-1, N = 2; toc1-101, N = 2; lux-4, N = 2. For all, n = 5–18. N represents the number of independent experiments and n the total number of organs tracked. See S1 File for exact n, test statistics, and percentage rhythmicity of each organ. Box plots indicate the median and upper and lower quartiles, and whiskers the 9th and 91st percentiles of organs scored as rhythmic. Individual data points are shown when the number of rhythmic plants is less than five. Horizontal position of scatter points is for clarity and has no meaning. Underlying data are available from https://gitlab.com/slcu/teamJL/greenwood_etal_2019. GI, GIGANTEA; LD, light-dark; LL, constant light; LUC, LUCIFERASE. (TIF) [file pbio.3000407.s012.tif]

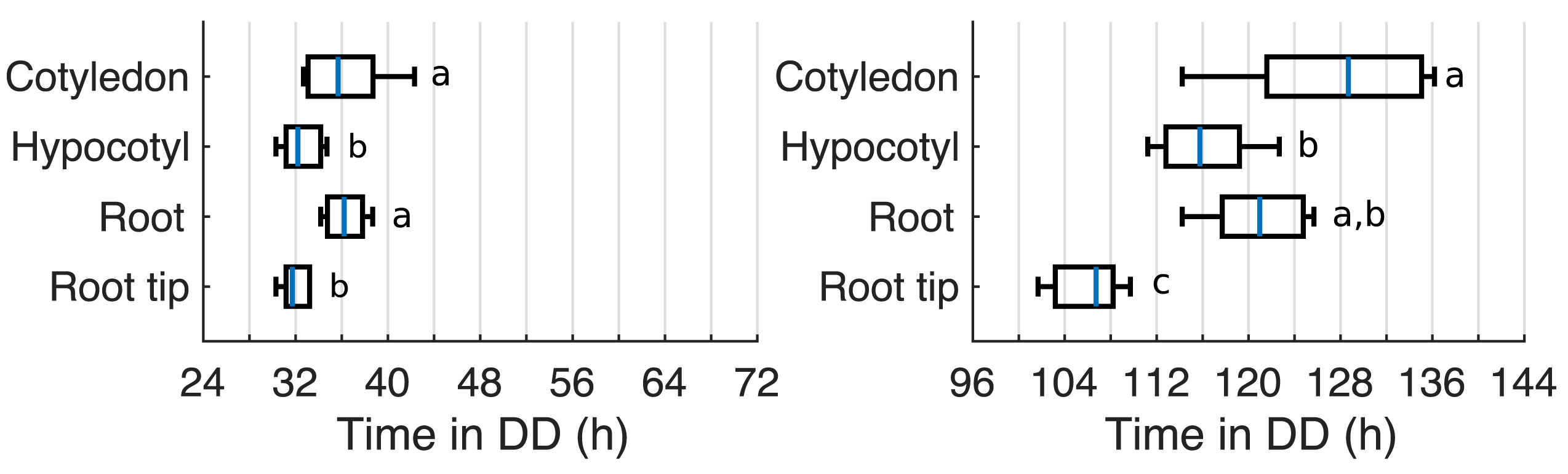

Supplement: S13 Fig — Times of peaks of GI::LUC expression in different organs during the first (left) and final (right) observed oscillations under DD. Means are statistically different (p < 0.05, one-way ANOVA, Tukey post hoc tests) if they do not have a letter in common. N and n are as presented in Fig 6. N represents the number of independent experiments and n the total number of organs tracked. See S2 File for exact n, test statistics, and percentage rhythmicity of each organ. Box plots indicate the median and upper and lower quartiles, and whiskers the 9th and 91st percentiles of organs scored as rhythmic. Underlying data are available from https://gitlab.com/slcu/teamJL/greenwood_etal_2019. DD, constant darkness. GI, GIGANTEA; LUC, LUCIFERASE. (TIF) [file pbio.3000407.s013.tif]

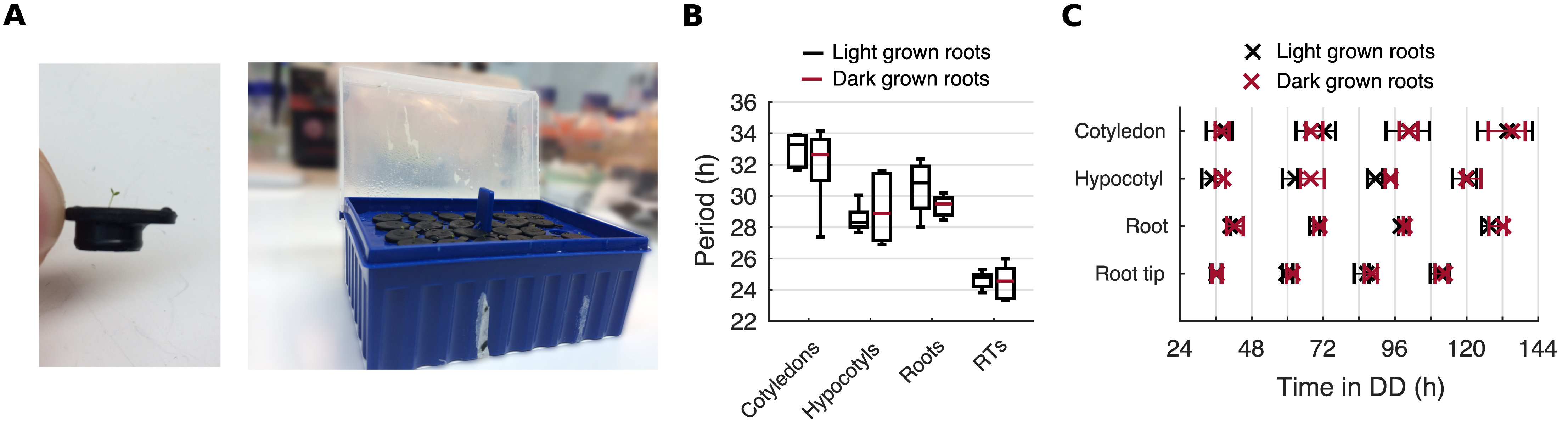

Supplement: S14 Fig — (A) Seeds are sown on agar-filled black micro-centrifuge tube lids with a piercing in the lid (left), and suspended in MS liquid in a floating micro-centrifuge tube rack (right), as described previously [88]. Seedlings are entrained for 4 d, with the roots either exposed to light or kept in the dark using this system. Seedlings are then imaged under DD. Note that images include a blur selectively on the background in order to highlight these components. (B) Period estimates of GI::LUC expression for the different organs when roots are exposed to light during entrainment or when kept in the dark. All comparisons between period estimates are not significant, p < 0.05, by two-tailed t test, Welch correction. Box plots indicate the median and upper and lower quartiles, and whiskers the 9th and 91st percentiles of organs scored as rhythmic. (C) Times of peaks of GI::LUC expression for the different organs presented in B. Plots represent the 25th percentile, median, and the 75th percentile for the peak times of organs scored as rhythmic. N = 3; n = 14–15. N represents the number of independent experiments and n the total number of organs tracked. See S1 and S2 Files for exact n, test statistics, and percentage rhythmicity of each organ. Underlying data are available from https://gitlab.com/slcu/teamJL/greenwood_etal_2019. DD, constant darkness; GI, GIGANTEA; LUC, LUCIFERASE; MS, Murashige and Skoog. (TIF) [file pbio.3000407.s014.tif]

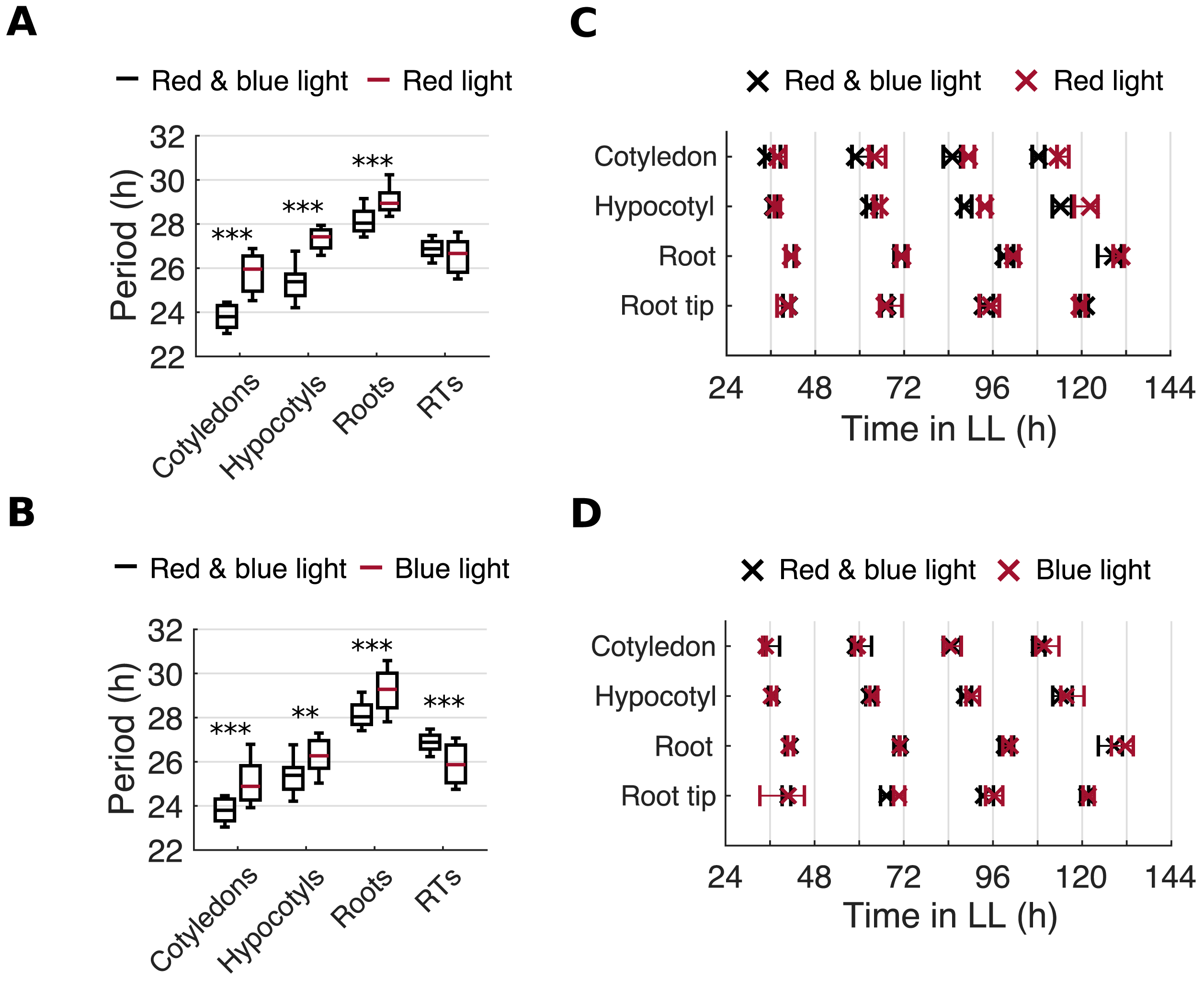

Supplement: S15 Fig — (A, B) Period estimates of GI::LUC expression for different organs under constant red and blue light, plotted against red-light-only (A) or blue-light-only data (B). ***p < 0.001, two-tailed t test, Welch correction. Box plots indicate the median and upper and lower quartiles, and whiskers the 9th and 91st percentiles of organs scored as rhythmic. (C, D) Times of peaks of GI::LUC expression in different organs imaged under constant red and blue light, plotted against constant red (C) or constant blue light data (D). Plots represent the 25th percentile, median, and the 75th percentile for the peak times of organs scored as rhythmic. For red and blue light data, N and n are as presented in Fig 2. For constant red light, N = 2; constant blue light, N = 2. For both, n = 22–25. N represents the number of independent experiments and n the total number of organs tracked. See S1 and S2 Files for exact n, test statistics, and percentage rhythmicity in each organ. Constant blue light data are an analysis of time-lapse movies we carried out in previous work [5]. Underlying data are available from https://gitlab.com/slcu/teamJL/greenwood_etal_2019. GI, GIGANTEA; LUC, LUCIFERASE. (TIF) [file pbio.3000407.s015.tif]

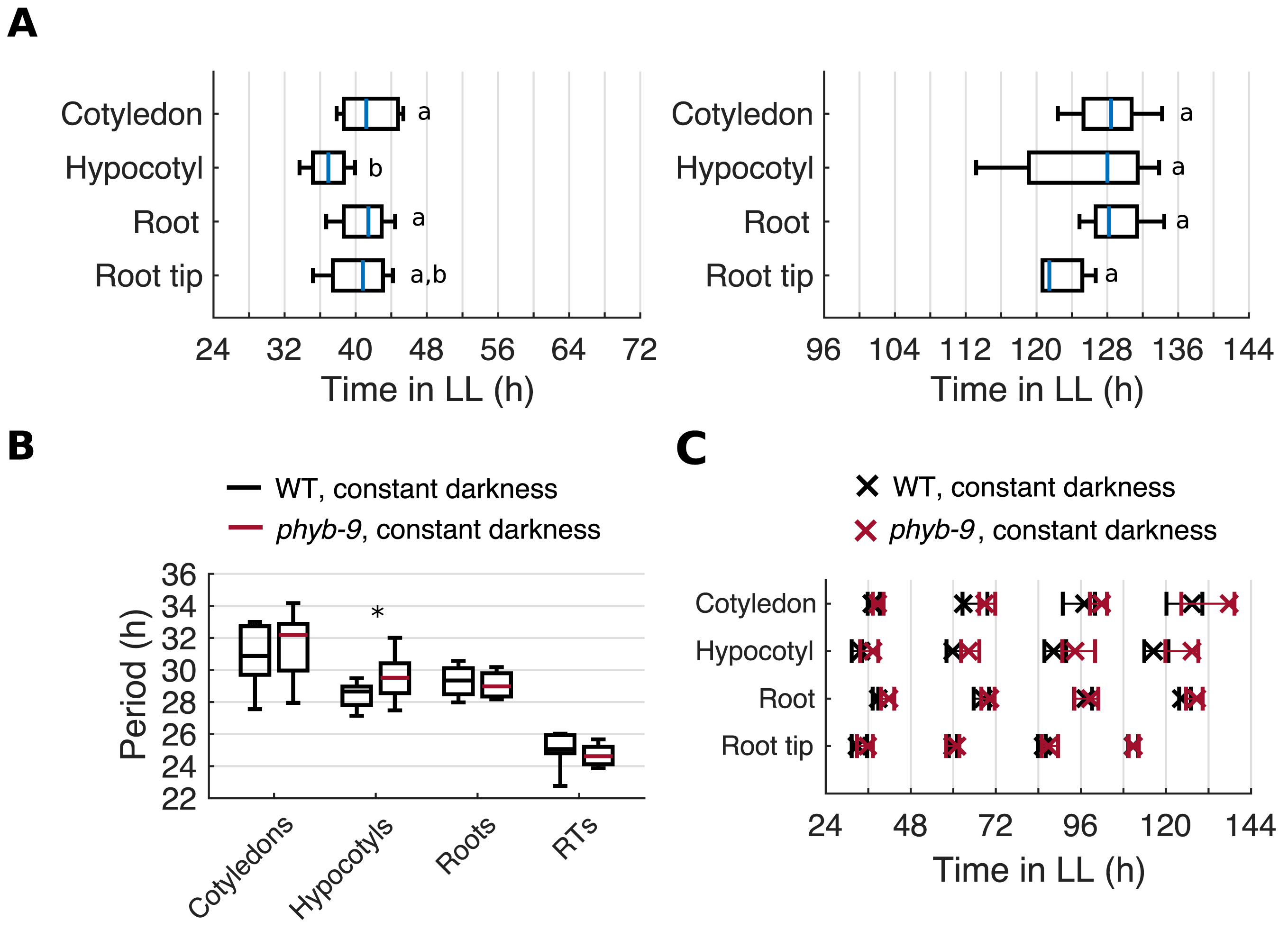

Supplement: S16 Fig — (A) Times of peaks of GI::LUC expression in different organs during the first (left) and final (right) observed oscillations in the phyb-9 mutant imaged under constant red light. Means are statistically different (p < 0.05, one-way ANOVA, Tukey post hoc tests) if they do not have a letter in common. (B) Period estimates of GI::LUC expression for different organs in the phyb-9 mutant imaged under DD. *p < 0.05, two-tailed t test, Welch correction. Box plots indicate the median and upper and lower quartiles, and whiskers the 9th and 91st percentiles of organs scored as rhythmic. (C) Times of peaks of GI::LUC expression in different organs in the phyb-9 mutant imaged under DD. For phyb-9 red light, N and n are as presented in Fig 6; phyb-9 DD, N = 2, n = 17–18. N represents the number of independent experiments and n the total number of organs tracked. See S1 and S2 Files for exact n, test statistics, and percentage rhythmicity of each organ. Plots represent the 25th percentile, median, and the 75th percentile for the peak times of organs scored as rhythmic. Underlying data are available from https://gitlab.com/slcu/teamJL/greenwood_etal_2019. DD, constant darkness; GI, GIGANTEA; LUC, LUCIFERASE; PHYB, PHYTOCHROME B. (TIF) [file pbio.3000407.s016.tif]

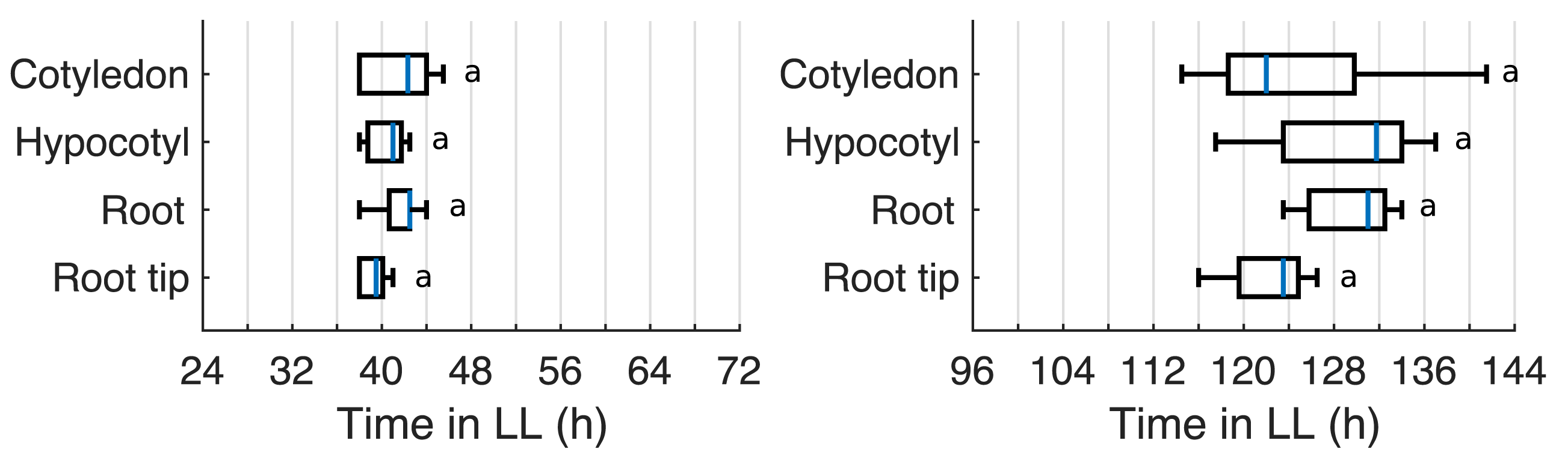

Supplement: S17 Fig — Times of peaks of GI::LUC expression in different organs during the first (left) and final (right) observed oscillations during the inhibition of photosynthesis by DCMU. Means are statistically different (p < 0.05, one-way ANOVA, Tukey post hoc tests) if they do not have a letter in common. N and n are as presented in Fig 7. N represents the number of independent experiments and n the total number of organs tracked. See S2 File for exact n, test statistics, and percentage rhythmicity of each organ. Box plots indicate the median and upper and lower quartiles, and whiskers the 9th and 91st percentiles of organs scored as rhythmic. Underlying data are available from https://gitlab.com/slcu/teamJL/greenwood_etal_2019. DCMU, 3-(3,4-dichlorophenyl)-1,1-dimethylurea; GI, GIGANTEA; LUC, LUCIFERASE. (TIF) [file pbio.3000407.s017.tif]

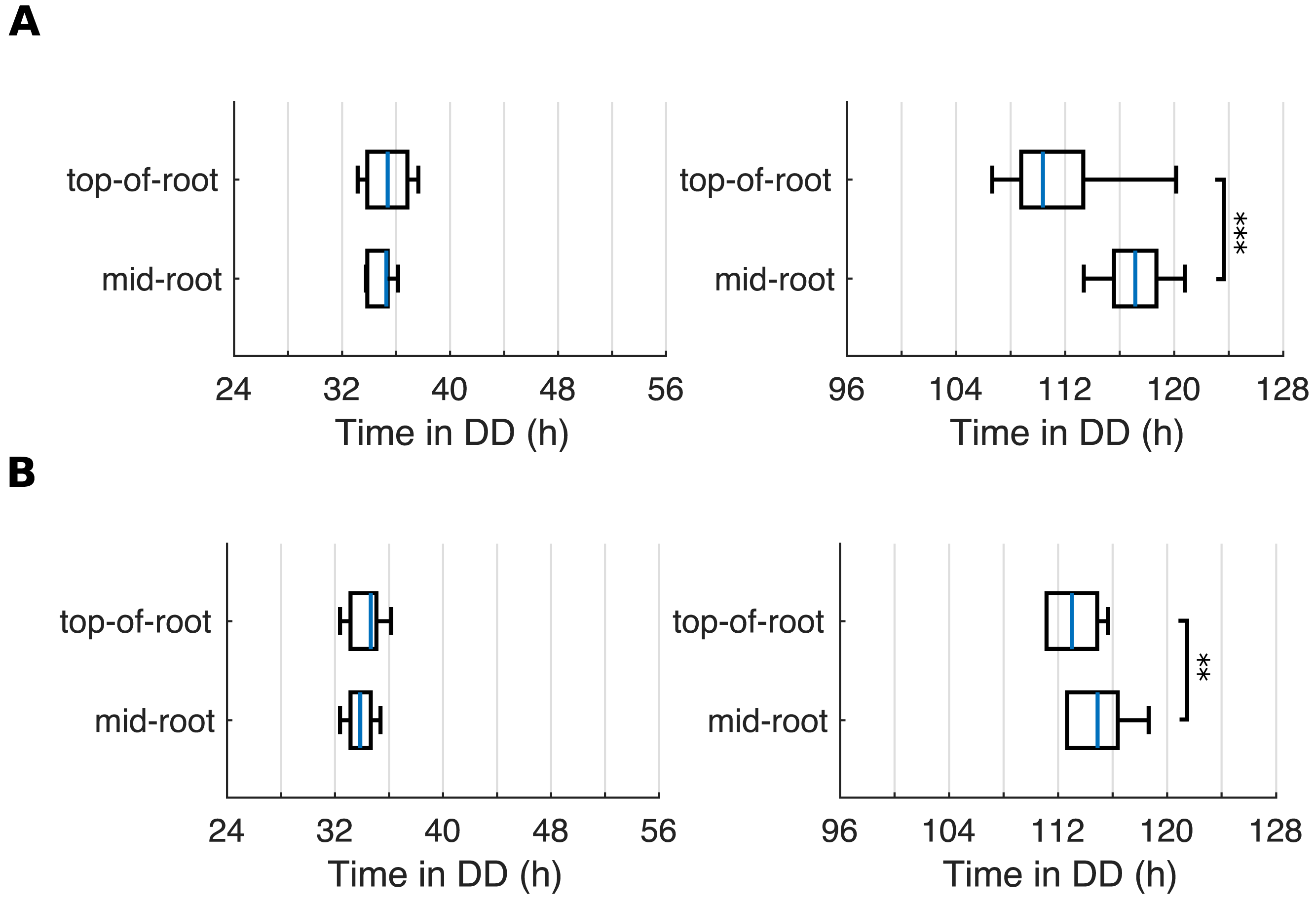

Supplement: S18 Fig — (A) Times of peaks of GI::LUC expression in different regions during the first (left) and final (right) observed oscillations during the partial contact of the root with sucrose. ***p < 0.001, Wilcoxon rank-sum test. (B) Times of peaks of expression in different regions during the first (left) and final (right) observed oscillations during the partial contact of the root with mannitol. **p < 0.01, Wilcoxon rank-sum test. N and n are as presented in Fig 7. N represents the number of independent experiments and n the total number of organs tracked. See S2 File for exact n, test statistics, and percentage rhythmicity of each organ. Box plots indicate the median and upper and lower quartiles, and whiskers the 9th and 91st percentiles of organs scored as rhythmic. Underlying data are available from https://gitlab.com/slcu/teamJL/greenwood_etal_2019. DD, constant darkness; GI, GIGANTEA; LUC, LUCIFERASE. (TIF) [file pbio.3000407.s018.tif]
